# Cynaropicrin disrupts tubulin and c-Myc-related signaling and induces parthanatos-type cell death in multiple myeloma

**DOI:** 10.1038/s41401-023-01117-3

**Published:** 2023-06-21

**Authors:** Joelle C. Boulos, Ejlal A. Omer, Daniela Rigano, Carmen Formisano, Manik Chatterjee, Ellen Leich, Sabine M. Klauck, Le-tian Shan, Thomas Efferth

**Affiliations:** 1 Department of Pharmaceutical Biology, Institute of Pharmaceutical and Biomedical Sciences, Johannes Gutenberg University, Staudinger Weg 5, 55128 Mainz, Germany; 2 Department of Pharmacy, University of Naples “Federico II”, Naples, Italy; 3 University Hospital Würzburg, Translational Oncology, Comprehensive Cancer Center Mainfranken, Würzburg, Germany; 4 Julius Maximilian University, Institute of Pathology, Würzburg, Germany; 5 Comprehensive Cancer Center Mainfranken, Translational Oncology, University Hospital of Würzburg, Würzburg, Germany; 6 Division of Cancer Genome Research, German Cancer Research Center (DKFZ), German Cancer Consortium (DKTK), National Center for Tumor Diseases (NCT), Heidelberg, Germany; 7 The First Affiliated Hospital, Zhejiang Chinese Medical University, Hangzhou, 310053 China

**Keywords:** hematological malignancies, multiple myeloma, cynaropicrin, c-Myc, microtubules, parthanatos, network pharmacology, xenograft tumor zebrafish model

## Abstract

The majority of blood malignancies is incurable and has unforeseeable remitting-relapsing paths in response to different treatments. Cynaropicrin, a natural sesquiterpene lactone from the edible parts of the artichoke plant, has gained increased attention as a chemotherapeutic agent. In this study, we investigated the effects of cynaropicrin against multiple myeloma (MM) cells in vitro and assessed its in vivo effectiveness in a xenograft tumor zebrafish model. We showed that cynaropicrin exerted potent cytotoxicity against a panel of nine MM cell lines and two leukemia cell lines with AMO1 being the most sensitive cell line (IC_50 _= 1.8 ± 0.3 µM). Cynaropicrin (0.8, 1.9, 3.6 µM) dose-dependently reduced c-Myc expression and transcriptional activity in AMO1 cells that was associated with significant downregulation of STAT3, AKT, and ERK1/2. Cell cycle analysis showed that cynaropicrin treatment arrested AMO1 cells in the G_2_M phase along with an increase in the sub-G_0_G_1_ phase after 24 h. With prolonged treatment times, cells accumulated more in the sub-G_0_G_1_ phase, implying cell death. Using confocal microscopy, we revealed that cynaropicrin disrupted the microtubule network in U2OS cells stably expressing α-tubulin-GFP. Furthermore, we revealed that cynaropicrin promoted DNA damage in AMO1 cells leading to PAR polymer production by PARP1 hyperactivation, resulting in AIF translocation from the mitochondria to the nucleus and subsequently to a novel form of cell death, parthanatos. Finally, we demonstrated that cynaropicrin (5, 10 µM) significantly reduced tumor growth in a T-cell acute lymphoblastic leukemia (T-ALL) xenograft zebrafish model. Taken together, these results demonstrate that cynaropicrin causes potent inhibition of hematopoietic tumor cells in vitro and in vivo.

## Introduction

Hematological malignancies are a group of cancerous tumors in which immunological or defective hematological cells fail to differentiate and perpetually proliferate, impairing the function of biological organisms [1]. They primarily fall into one of three categories: lymphoma, multiple myeloma (MM), and leukemia [2]. Hematological malignancies are among the most fatal diseases, posing a major threat to human health and life due to their high mortality rate. In fact, hematological malignancies are unique in that they cannot be surgically extirpated as solid tumors, and their clinical first-line therapies mostly consist of chemotherapy, radiation, and hematopoietic stem cell transplantation [3]. Even though standard first-line medicines have some effect, the general efficacy is unsatisfactory because of relapses and refractory conditions brought on by the emergence of primary and secondary drug resistance [4].

T-cell acute lymphoblastic leukemia (T-ALL) emanates from genetic abnormalities that assemble in the course of T-cell maturation in the thymus, leading to differentiation arrest and abnormal spread of immature progenitors [5]. Although T-ALL survival rates have significantly increased over the past 50 years, relapsed and refractory patients are still very difficult to treat, and the prognosis for those who cannot withstand intense treatment is still bleak [6].

MM is a hematological plasma cell malignancy that comprises around 10% of all blood cancers [7]. The median survival rate for stage III MM patients is about 29 months, whereas stage I and stage II patients survive almost 62 and 44 months, respectively [8]. Given the increase in MM incidence as well as the mortality rate, several anti-cancer therapies have been established, e.g., targeted drug therapies (immunomodulatory drugs—IMiDs, proteasome inhibitors—PIs, monoclonal antibodies—mAbs-, etc.), combination therapies based on lenalidomide, bortezomib, and others, radiotherapy, stem cell transplantation, corticosteroids, and bisphosphonate treatment [9]. Even though these therapies protracted the anticipated life span of MM patients, many concerns have been raised regarding the drawbacks resulting from the prolonged application of these treatments.

As scientists became aware of the imminent side effects of the current hematologic therapies, they searched for natural products (NPs) as a possible replacement. Plant-based diets, focusing on the consumption of not only vegetables and fruits, but also whole grains, are inversely associated with the incidence of malignancies, as they are rich in phytochemicals [10]. Over the past decades, the application of phytochemicals as adjuvant anti-neoplastic agents has been a rising trend [11], because NPs display the following main characteristics: (i) “metabolite-likeness”, (ii) active transport metabolites, and (iii) high bioavailability [12–14]. Hence, studies on NPs skyrocketed in the past three decades [15]. Numerous phytochemicals have been shown to exhibit cell cycle arrest, apoptosis, anti-angiogenesis, and miRNA modulation in MM. Additionally, agaricus, curcumin, and neovastat have been applied in clinical trials for the treatment of MM [16–18]. Altogether, these evidences shed light on the potency of NPs and their bioactive compounds in MM treatment [9]. Sesquiterpene lactones (SQL) constitute one of the major and most studied class of plant-derived phytochemicals [19], as they have potent anti-inflammatory, anti-tumor, antibiotic, phyto-toxic, insect-feeding deterrent, and schistosomicidal potential bioactivities [20].

**Fig. 1:**
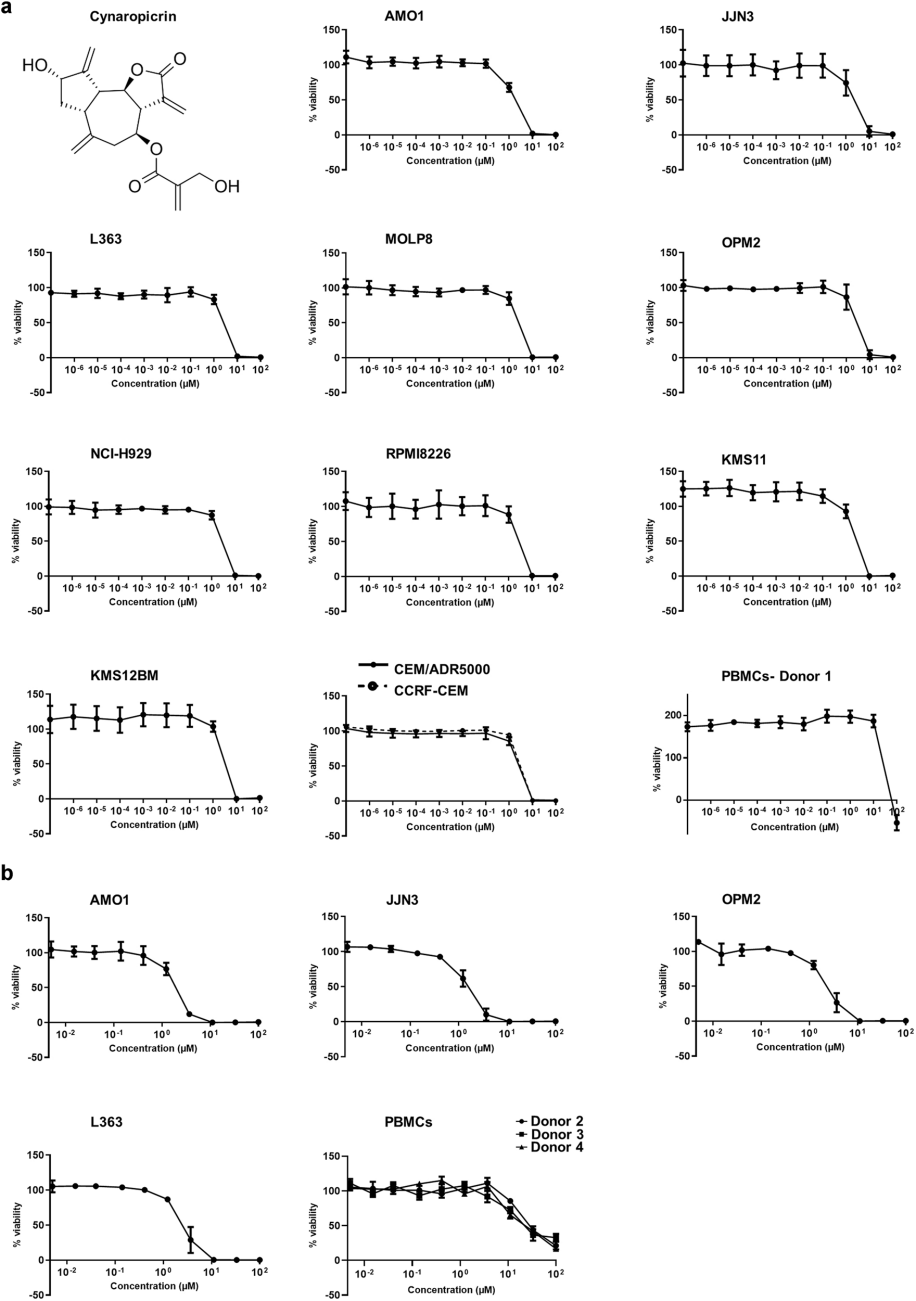
Chemical structure of the lipophilic compound cynaropicrin and the cytotoxic effect of cynaropicrin in nine different human multiple myeloma (MM) cell lines (AMO1, KMS11, JJN3, MolP8, L363, NCI-H929, RPMI8226, OPM2, and KMS12BM), in human AML cells (CCRF/CEM, CEM/ADR5000), and PBMCs. Each concentration is obtained by calculating the mean value ± SD of three experiments performed at different time points with six replicates in each experiment. **a** Using a wide concentration range ten-fold apart from 10^−7^ to 10^2^, **b** Using a narrower concentration range three-fold apart from 10^−2.35^ to 10^2^.

Cynaropicrin, a SQLs of the guaianolide type, has a γ-butyrolactone ring, a crucial pharmacophore that is implicated in several biological activities (Fig. 1). Cynaropicrin was first isolated from the edible artichoke plant (*Cynara scolymus* L.) [21], and it is currently regarded as a chemotaxonomic marker of the artichoke plant [22]. The bitter taste of artichokes is associated with elevated SQL amounts, particularly of cynaropicrin, which causes around 80% of the typical bitter taste of artichoke plants [23]. Besides, cynaropicrin has been isolated from numerous species of the genus *Centaurea* and *Saussurea* [24–28], and it has several biological and pharmacological properties such as anti-inflammatory, anti-cancer, anti-parasitic, anti-protozoal, anti-hyperlipidemic, anti-hepatitis C viral, anti-photoaging, anti-spasmodic, anti-feedant activities, and is also an inhibitor of NF-κB and activator of bitter sensory receptors [29]. Several studies reported the effect of cynaropicrin against various cancer types. Cynaropicrin induced apoptosis and weak G2/M arrest in a human gastric adenocarcinoma cell line [30]. Cynaropicrin was also investigated for melanoma and thyroid cancer [31, 32], triple-negative breast cancer [33], cervical cancer [34], colorectal cancer [35], and lymphoma and leukemia, especially multidrug-resistant cell lines [24, 36].

However, no studies have yet been performed to examine the response of MM to cynaropicrin in vitro or to evaluate the in vivo antiproliferative activity of cynaropicrin through a leukemia model. Therefore, the aim of this project was to investigate the effect of cynaropicrin on MM cancer cells in vitro and to assess the in vivo effectiveness of cynaropicrin using a T-ALL zebrafish model.

## Materials and methods

### Cell lines

The drug-sensitive CCRF-CEM T-ALL and their counterpart multidrug-resistant P-glycoprotein-overexpressing CEM/ADR5000 leukemia cells were provided by Dr. Axel Sauerbrey (Children’s Hospital, University of Jena, Germany). MM cell lines (AMO1, JJN3, KMS12BM, KMS11, L363, MolP8, NCI-H929, OPM2) were kindly supplied by Dr. Manik Chatterjee and Dr. Ellen Leich (University of Würzburg, Germany). RPMI8226 cells were obtained from the American Type Cell Culture Collection (ATCC^®^ CCL-155™, USA). All MM cells were mycoplasma free. MM and T-ALL cells were cultured in RPMI-1640 (Life Technologies, Darmstadt, Germany) supplemented with 10% FBS (Life Technologies) and 1% penicillin (1000 U/mL)/streptomycin (100 μg/mL) (Life Technologies). Cells were kept in a 5% CO_2_ incubator at 37 °C.

Human HEK293 embryonic kidney cells were kindly provided by Prof. Dr. Christina Friedland (Johannes Gutenberg University, Germany) and bone osteosarcoma U2OS human cells stably expressing α-tubulin-GFP protein were kindly provided by Dr. Joachim Hehl (Light microscope center, ETH Zurich). HEK293 and U2OS cells were cultured in DMEM (Life Technologies) supplemented with 10% FBS (Life Technologies) and 1% penicillin (1000 U/mL)/streptomycin (100 μg/mL) (Life Technologies). Cells were kept in a 5% CO_2_ incubator at 37 °C.

Fresh blood samples were collected from four healthy donors at the Department of Hematology, Oncology, and Pneumology (University Medical Center of the Johannes Gutenberg University, Mainz, Germany) and flowed in plastic Monovette EDTA tubes. Histopaque^®^ (Sigma-Aldrich, Taufkirchen, Germany) was used to isolate human peripheral blood mononuclear cells (PBMCs). In brief, 3 mL of fresh blood were thoroughly layered on top of Histopaque® and centrifuged for 30 min at 400 × *g* and 4 °C. Subsequently, the buffy coat consisting of PBMCs was separated, washed with PBS, and subjected to centrifugation at 250 × *g* thrice, for 10 min each. The cell pellet was suspended in Panserin 413 growth media (PAN-Biotech, Aidenbach, Germany) supplemented with 2% phytohemagglutinin M (PHA-M, Life Technologies).

### Cell viability assay

The resazurin reduction assay was applied to determine the sensitivity of leukemia, MM cell lines, and PBMCs to cynaropicrin. Briefly, 10^4^ cells/well were seeded in a flat bottom 96-well plate. Cells were immediately treated with different cynaropicrin concentrations for 72 h.

AMO1 cells (10^4^ cells/well) were also seeded in a flat bottom 96-well plate. Cells were immediately treated with 1.8, 3.6, and 7.2 µM of cynaropicrin as well as 1.8, 3.6, and 7.2 µM of cynaropicrin in combination with the PARP inhibitor PJ34 (10 µM) (528150, Sigma–Aldrich, Darmstadt, Germany), or the caspase inhibitor z-vad-fmk (50 µM) (627610, Sigma–Aldrich, Darmstadt, Germany), or the autophagy inhibitor Bafilomycin A1 (50 nM) (J61835.MCR, Thermo Fisher Scientific, Dreieich, Germany), or the autophagy inducer Rapamycin (50 nM) (J62473.MF, Thermo Fisher Scientific, Dreieich, Germany) for 48 h. The 96-well plates were then incubated at 37 °C and 5% CO_2_ for 4 h with resazurin. The reduction of resazurin by living cells generated a fluorescence, which was detected with an Infinite M2000 Pro plate reader (Tecan, Crailsheim, Germany) at an excitation wavelength of *λ*/nm = 544 and an emission wavelength of *λ*/nm = 590. Later on, GraphPad Prism 5 software (GraphPad Software, San Diego, CA) was used to plot the cell viability against the concentration of cynaropicrin and to determine the IC_50_ values from three independent experiments with six replicates each [37].

### Gene expression profiles

AMO1 cells were treated with 1.8 µM of cynaropicrin or with DMSO. After 24 h treatment, RNA extraction was performed using the RNeasy Kit from Qiagen (Hilden, Germany). As previously described, gene expression profiling was obtained by microarray hybridization of duplicate samples using Affymetrix Clariom S human chips (Affymetrix, Santa Clara, CA, USA) at the Genomics and Proteomics Core Facility of the German Cancer Research Center (DKFZ, Heidelberg) [38].

### Real-time quantitative polymerase chain reaction (RT-PCR)

Luna Script™ RT SuperMix Kit (E3010) (New England Biolabs GmbH, Frankfurt, Germany) was used to convert 1 μg of the extracted RNA into cDNA following the manufacturer’s instruction. 5 × Hot Start Taq EvaGreen^®^ qPCR Mix (Axon-Labortechnik, Kaiserslautern, Germany) was used to carry out RT-qPCR following manufacturer’s instruction. The Primer-BLAST tool was applied to design the primers (Table 1), which were double-checked for rightness with the Oligo Analyze Tool from Eurofins Genomics Germany GmbH (Ebersberg, Germany) and purchased from Eurofins Genomics. *GAPDH* was chosen as a reference gene. RT-qPCR was performed using CFX384™ Real-Time PCR Detection System (Bio-Rad Laboratories GmbH, Feldkirchen, Germany). Each sample was measured three times, each time in duplicates. The fold change FC was determined based on the (Ct) of the gene of interest, represented as (gene), and the reference gene represented as (ref) obtained from the control and the sample [39].

$$\varDelta {{{{{{\rm{C}}}}}}}_{{{{{{\rm{t}}}}}}}={{{{{{\rm{C}}}}}}}_{{{{{{\rm{t}}}}}}({{{{{\rm{gene}}}}}})}-{{{{{{\rm{C}}}}}}}_{{{{{{\rm{t}}}}}}({{{{{\rm{ref}}}}}})}$$

$$\varDelta \varDelta {{{{{{\rm{C}}}}}}}_{{{{{{\rm{t}}}}}}}=\varDelta {{{{{{\rm{C}}}}}}}_{{{{{{\rm{t}}}}}}({{{{{\rm{sample}}}}}})}-\varDelta {{{{{{\rm{C}}}}}}}_{{{{{{\rm{t}}}}}}({{{{{\rm{control}}}}}})}$$

$${{{{{\rm{FC}}}}}}={\log }_{2}({2}^{-\varDelta \varDelta {{{{{\rm{Ct}}}}}}})$$

**Table 1:** Design of the primer’s sequences (5′→3′) for RT-PCR.

Genes	Forward primer	Reverse primer
*STAT3*	TCT GTG TGA CAC CAA CGA CC	GGA CTC AAA CTG CCC TCC TG
*MAP2K2*	TTG TGA ACG AGC CAC CTC C	TGA GCA TCT TCA GGT CCG C
*AKT1*	GCG GCA GGA CCG AGC	CGC CTG CTC CCG TCT TC
*c-MYC*	CTT CTC TCC GTC CTC GGA TTC T	GAA GGT GAT CCA GAC TCT GAC CTT
*GAPDH*	GCT CTC TGC TCC TCC TGT TC	GAC TCC GAC CTT CAC CTT CC

### Cell cycle analysis

The cell cycle perturbations of AMO1 cells were studied using Propidium iodide (PI) 24, 48, and 72 h post-treatment with different cynaropicrin concentrations (0.5, 0.9, 1.8, and 3.6 µM) or media alone. Cells were then harvested and fixed with 80% ethanol at −20 °C. After each specific incubation period, cells were incubated in PI staining solution (Thermo Fisher Scientific, Dreieich, Germany) at 4 °C. Fifteen minutes later, PI staining was measured using an Accuri C6 flow cytometer (Becton-Dickinson, Heidelberg, Germany). Total DNA content was detected on FL2-A [38].

### Fluorescence confocal microscopy of the microtubule organization

Human osteosarcoma U2OS cancer cells stably transfected with an α-tubulin-GFP construct were cultured in a sterile µ-Slide 8 Well (ibidi, Gräfelfing, Germany). Aliquots of 30,000 cells/well were left overnight in an incubator at 37 °C/5 % CO_2_ to attach, then cells were treated with 1.8 or 3.6 µM of cynaropicrin or DMSO (negative control) or vincristine (1 µM), a polymerization inhibitor, or paclitaxel (1 µM), a depolymerization inhibitor, for 24 h. Later, cells were rinsed with PBS and fixed for 15 min with 4% paraformaldehyde. After fixation, cells were washed three times with PBS and the nucleus of each cell was stained for 5 min with 1 µg/mL 4′,6-diamidino-2-phenylindole (DAPI) (Sigma–Aldrich, Darmstadt, Germany) at room temperature. Afterward, cells were rinsed three times with PBS to get rid of excessive DAPI and ibidi mounting medium (ibidi, Gräfelfing, Germany) was added to each slide. Fluorescence images were obtained using an AF7000 widefield fluorescence microscope (Leica Microsystems, Wetzlar, Germany). The blue laser (*λ*/nm = 470) was used to excite both GFP and DAPI. The emission wavelength of GFP is *λ*/nm = 525, however the emission wavelength of DAPI is *λ*/nm = 447. Fluorescence images were analyzed with Fiji ImageJ software (National Institutes of Health, Bethesda, MD, USA) [40].

AMO1 cells were seeded in a six-well plate (500,000 cells/well) and treated with different concentrations of cynaropicrin (1.8 or 3.6 µM) or with DMSO for 24 h at 37 °C/5 % CO_2_. Cells were then harvested, washed with HBSS, and cytospinned over slides (Thermo Fisher Scientific, Dreieich, Germany) for 5 min at 123 × *g*. Afterwards, cells were stained with Tubulin Tracker™ Deep Red (Thermo fisher Scientific, Dreieich, Germany) for 30 min at 37 °C and 5% CO_2_. Cells were then washed with HBSS thrice for 5 min each. Cells nuclei were stained with Hoechst 33342 Nuclear Stain (H3570, Thermo Fisher Scientific, Darmstadt, Germany) for 30 min at room temperature. After 30 min, cells were washed with HBSS thrice for 5 min each and polymerized tubulin in AMO1 cells was imaged at 40 × magnification using an AF7000 widefield fluorescence microscope.

### Apoptosis examination

Apoptosis was examined using a Ratiometric Membrane Asymmetry Probe/Dead Cell Apoptosis Kit (Thermo Fisher Scientific, Darmstadt, Germany). A violet excitable dye named 4′-*N*, *N*-diethylamino-6-(*N*,*N*,*N* dodecyl-methylamino-sulfopropyl)-methyl-3-hydroxyflavone (F2N12S) is used to detect changes in membrane asymmetry through apoptosis [41]. Briefly, AMO1 cells were seeded in a 6-well plate (1 × 10^6^ cells/well) and treated with different concentrations of cynaropicrin (0.5, 0.9, 1.8, and 3.6 µM) or with DMSO, used as a negative control, for 48 h at 37 °C/5 % CO_2_. Cells were then harvested and washed with HBSS (Hanks Balanced Salt Solution) twice. Consecutively, cells were incubated with F2N12S at a final concentration of 200 nM and SYTOX™ AADvanced™ dead cell stain at a final concentration of 1 µM for 5 min, in the dark, at room temperature. Cells were analyzed with a BD LSRFortessa SORP (Becton Dickinson, Heidelberg, Germany) flow cytometer. F2N12S was excited by a violet laser (*λ*/nm = 405) and the emission light was assembled with a 585/15 bandpass filter representing the orange fluorescence channel and 530/30 bandpass filter representing the green fluorescence channel (The ratio parameter (585/530) was set up in the software). SYTOX™ AADvanced™ dead cell stain was excited by a blue laser (λ/nm = 488) and the emission light was assembled with a 670/30 bandpass filter as suggested by the manufacturer [42]. Cells were then gated using the F- (forward) and S- (side) scatters. Singlets were selected by gating according to the *A*- (area) and *W*- (width) scatters. From each well, 10^4^ events from the first gate (FSC/SSC) were recorded. Data were then analyzed with FlowJo V10.6.2 software (Becton Dickinson).

### Mitochondrial membrane potential (MMP)

MMP was measured using the JC-1 mitochondrial membrane potential assay kit (Cayman Chemical, Ann Arbor, Michigan, United States). Briefly, 10^5^ AMO1 cells/well were seeded in a flat bottom 96-well plate. Cells were treated with different concentrations of cynaropicrin (0.5, 0.9, 1.8, 3.6, or 7.2 µM) or DMSO (negative control), or 1 µM of bortezomib (positive control) for 48 h at 37 °C/5 % CO_2_. Cells were then incubated with 10 µL of JC-1 solution for 15 min at 37 °C/5 % CO_2_. Afterwards, cells were centrifuged at 277 × *g* for 5 min and washed twice with 200 µL assay buffer. Finally, cells were re-suspended in 200 µL assay buffer and JC-1 staining was measured using a BD LSR Fortessa SORP flow cytometer. J-aggregates (living cells) were excited by a yellow-green laser (*λ*/nm = 561) and the emission light was assembled with a 586/15 bandpass filter. JC-1 monomers (dead cells) were excited by a blue laser (*λ*/nm = 488) and the emission light was assembled with a 530/30 bandpass filter [43]. If JC-1 accumulates in healthy mitochondria, it exhibits red fluorescence, however when it accumulates in damaged mitochondria, it emits green fluorescence. Cells were then gated using the F- (forward) and S- (side) scatters. Singlets were selected by gating according to the *A*- (area) and *H*- (height) scatters. Data were then analyzed with FlowJo V10.6.2 software (Becton Dickinson).

### Western blot analyses

AMO1 cells were seeded in a six-well plate (1 × 10^6^ cells/well) and treated with different concentrations of cynaropicrin (0.9, 1.8, or 3.6 µM) or with DMSO, used as a negative control, for 48 h at 37 °C/5% CO_2_. Cells were then harvested and washed with PBS twice. To extract total proteins, cells were incubated in a 500 rpm shaker with M-PER^®^ Mammalian Protein Extraction Reagent supplemented with 1% Halt™ Protease Inhibitor Cocktail (Thermo Scientific, Frankfurt, Germany) for 30 min at 4 °C. Later, the cells were centrifuged at 277 × *g* for 15 min at 4 °C and the supernatant containing proteins was collected. NE-PER Nuclear and Cytoplasmic Extraction reagent (Thermo Fisher Scientific, Dreieich, Germany) was used to extract nuclear and cytoplasmic proteins following the manufacturer’s instructions. Protein concentration was determined with a NanoDrop 1000 spectrophotometer (Thermo Scientific, Frankfurt, Germany). Subsequently, 30 µg of the lysate was loaded to each lane of a 10% SDS-PAGE gel. Following the separation step, the proteins were transferred on a polyvinylidene difluoride membrane. The membrane was blocked with a blocking buffer consisting of 5% BSA in TBST for 1 h at room temperature. After the blocking step, the membranes were incubated overnight at 4 °C with primary antibodies (1:1000) against c-Myc (#9402), Akt (pan) (C67E7) (#4691), phospho-Akt (Ser473) (D9E) XP^®^ (#4060), p44/42 MAPK (Erk1/2) (137F5) (#4695), Phospho-p44/42 MAPK (Erk1/2) (Thr202/Tyr204) (D13.14.4E) XP^®^ (#4370), Stat3 (D3Z2G) (#12640), Phospho-Histone H2A.X (Ser139) (20E3) (#9718), PARP (#9542), AIF (D39D2) XP^®^ (#5318), caspase 7 (D2Q3L) (#12827), FoxO1 (C29H4) (#2880), GAPDH (D16H11) XP^®^ (#5174), β-Actin (13E5) (#4970) which were purchased from Cell Signaling Technology (Frankfurt a. M., Germany), P62 (18420-1-AP), Lamin B1 (66095-1-Ig), Beclin 1 (11306-1-AP), and Caspase 3/p17/p19 (19677-1-AP) which were purchased from Proteintech (Planegg-Martinsried, Germany) and finally Anti-PAR (AM80- 100UG) which was purchased from Merck (Darmstadt, Germany). Membranes were then washed thrice with TBST for 10 min each. Following the washing step, the membranes were incubated with either anti-rabbit IgG secondary antibody coupled to horseradish peroxidase (HRP-linked; 1:1000) (#7074S, Cell Signaling Technology, Frankfurt a. M., Germany) or anti-mouse IgG secondary antibody coupled to horseradish peroxidase (HRP-linked; 1:1000) (#7076S, Cell Signaling Technology, Frankfurt a. M., Germany) for 1 h [44]. Finally, membranes were incubated with Luminata™ Classico Western HRP substrate (Merck Millipore, Darmstadt, Germany) for 3 min and imaged using an Alpha Innotech FluorChem Q system (Biozym, Oldendorf, Germany). Protein expression was calculated using ImageJ software [45].

### Fluorescence microscopy of apoptosis inducing factor (AIF)

AMO1 cells were treated with different concentrations of cynaropicrin (0.9, 1.8, and 3.6 µM) and DMSO (negative control) for 48 h at 37 °C/5% CO_2_. After 48 h treatment, cells were harvested and washed with PBS. Cells were then cytospinned on microscope slides (Thermo Fisher Scientific, Dreieich, Germany) at a speed of 123 × *g* for 5 min. Subsequently, 4% paraformaldehyde was added on top of the cells for 15 min at room temperature to fix them. After fixation, cells were washed thrice with PBS, permeabilized with 1% Triton X-100 in PBS for 10 min at room temperature, and again washed thrice with PBS. Afterwards, cells were blocked with blocking buffer (1% BSA + 10% FBS in PBS) for 1 h. After blocking, the primary antibody AIF (D39D2) XP^®^ (#5318) (Cell Signaling Technology, Frankfurt a. M., Germany) was added to the microscope slides which were incubated at 4 °C in a humidified chamber overnight. The slides were washed thrice with PBS. After the washing step, slides were incubated with antirabbit secondary antibody-Alexa Fluor^®^ 488 Conjugate (Cell Signaling Technology) at room temperature in a dark humidified chamber. After 2 h, slides were rinsed three times with PBS. Cell nuclei were stained with 1 µg/mL of DAPI (Sigma–Aldrich, Darmstadt, Germany) for 5 min and slides were washed with PBS thrice. Finally, cells were mounted with Fluoromount-G^®^ (SouthernBiotech, Birmingham, AL, USA) and an AF7000 widefield fluorescence microscope (Leica Microsystems, Wetzlar, Germany) was used for visualization. Alexa Fluor® 488 was excited at *λ*/nm = 470 and emitted light at *λ*/nm = 525. DAPI was excited at *λ*/nm = 470 and emitted light at λ/nm = 447 [46].

### Cignal Myc reporter assay

c-Myc activity was determined using the cignal Myc reporter assay kit (CCS-012L) from Qiagen (Germantown, MD, USA). Myc reporter assay is made to keep track of the Myc signaling pathway’s activity in cells. A transfection-ready expression vector for c-Myc and a reporter vector for luciferase are included in the kit. As a first step, HEK293 cells were transfected with a c-Myc-luciferase reporter construct and cultured according to the manufacturer’s suggestions. HEK293 cells were used as hematopoietic cells are difficult to transfect. Cells were then treated with different concentrations of cynaropicrin or DMSO (negative control), or the known Myc inhibitor (10058-F4) (positive control) for 48 h. The activity of the c-Myc promoter was quantified using the Dual-glo^®^ Luciferase Reporter Assay System (E2920, Promega, Madison, WI, USA). An Infinite M2000 Pro™ plate reader (Tecan, Germany) was used to measure the luminescence of firefly and renilla luciferases [47].

$$	{{{{{\rm{c}}}}}}-{{{{{\rm{Myc}}}}}}\,{{{{{\rm{activity}}}}}} = \\ 	\quad({{{{{\rm{firefly}}}}}}\,{{{{{\rm{luciferase}}}}}}\,{{{{{\rm{luminescence}}}}}}/{{{{{\rm{renilla}}}}}}\,{{{{{\rm{luciferase}}}}}}\,{{{{{\rm{luminescence}}}}}})$$

$$	{{{{{\rm{Relative}}}}}}\,{{{{{\rm{luciferase}}}}}}= \\ 	\quad100\times ({{{{{\rm{firefly}}}}}}\,{{{{{\rm{luciferase}}}}}}\,{{{{{\rm{luminescence}}}}}}/{{{{{\rm{renilla}}}}}}\,{{{{{\rm{luciferase}}}}}}\,{{{{{\rm{luminescence}}}}}})$$

$$	{{{{{\rm{Normalized}}}}}}\,{{{{{\rm{c}}}}}}-{{{{{\rm{Myc}}}}}}\,{{{{{\rm{activity}}}}}}= \\ 	\quad{{{{{\rm{relative}}}}}}\,{{{{{{\rm{luciferase}}}}}}}_{({{{{{\rm{sample}}}}}})}/{{{{{\rm{relative}}}}}}\,{{{{{{\rm{luciferase}}}}}}}_{({{{{{\rm{DMSO}}}}}})}$$

### Xenograft zebrafish model

Adult wild-type AB strain zebrafish was ordered from the China Zebrafish Resource Center, Institute of Hydrobiology, China Academy of Science (Wuhan, China) and officially authorized by the Association for Assessment and Accreditation of Laboratory Animal Care International (SYXK 2012-0171). After 48 h of fertilization, natural pair-mating gave rise to zebrafish larvae which were kept in an aquaculture facility with an alternate photoperiod of 14 h day/10 h night. Zebrafish larvae were fed with one portion of fry flakes and two portions of live brine shrimps per day.

To initiate a T-ALL tumor xenograft zebrafish model, CCRF-CEM cells were stained with red fluorescence CM-Dil (1:1000). After 48 h of fertilization, 200 cells/fish were microinjected in the zebrafish larvae yolk sac. After 24 h of tumor growth, a fluorescent microscope (AZ100, Nikon, Tokyo, Japan) was used to verify the model. The CCRF-CEM cell-bearing zebrafish were then treated with different concentrations of cynaropicrin, and cis-platinum, used as positive control, respectively, for 24 h (*n* = 5 zebrafishes/group). The fluorescence intensity (Fi) of CCRF-CEM cell mass in every zebrafish was determined and the inhibitory rate was calculated as follows [48]:

$$	{{{{{\rm{inhibitory}}}}}}\,{{{{{\rm{rate}}}}}}( \% )= \\ 	\quad[1{-}({{{{{\rm{Fi}}}}}}\,{{{{{\rm{of}}}}}}\,{{{{{\rm{treated}}}}}}\,{{{{{\rm{group}}}}}}/{{{{{\rm{Fi}}}}}}\,{{{{{\rm{of}}}}}}\,{{{{{\rm{negative}}}}}}\,{{{{{\rm{control}}}}}}\,{{{{{\rm{group}}}}}})]\times 100 \%$$

### Statistical analysis

The results were presented as mean ± S.D. Statistics were calculated using the unpaired two-tailed Student’s *t*-test (Microsoft Excel, 2019). Significant results were those with a *P*-value less than 0.05.

## Results

### Cynaropicrin reduced MM and leukemia cells viability

The cytotoxic effect of cynaropicrin was analyzed using the resazurin reduction assay in MM, drug-sensitive and multidrug-resistant P-glycoprotein-overexpressing leukemia cells, as well as PBMCs. A significant obstacle in cancer treatment is the emergence of multidrug resistance (MDR) to chemotherapy. We have previously identified lead compounds in the early phases of drug discovery that are not substrates of the ATP-binding cassette (ABC) transporters [49]. We have also demonstrated that natural products may provide attractive lead molecules for the generation of collateral sensitive anticancer compounds [50]. In this context, we decided to study the cytotoxicity of cynaropicrin against CEM/ADR5000 cells.

Cynaropicrin exhibited a strong cytotoxicity in all tested MM cells after 72 h of treatment, with AMO1 being the most sensitive cell line (IC_50_ = 1.8 ± 0.3 µM) and KMS12BM being the least sensitive (IC_50_ = 3.2 ± 0.2 µM) (Fig. 1a, Table 2). Moreover, the degree of responsiveness of CCRF-CEM (IC_50_ = 2.9 ± 0.0 µM) and CEM/ADR5000 (IC_50_ = 2.6 ± 0.2 µM) to cynaropicrin was comparable. The IC_50_ values of AMO1, JJN3, OPM2, and L363 obtained with a narrow concentration range (three-fold apart from 100 µM to 0.005 µM) (Fig. 1b, Table 3) were comparable with the IC_50_ values obtained with a wide concentration range (ten-fold apart from 100 µM to 10^−6^ µM) (Fig. 1a, Table 2) with a correlation coefficient (*R*^2^) equal to 0.89, indicating that the IC_50_ values obtained with the wide concentration range were accurate.

**Table 2:** IC_50_ values of cynaropicrin in MM, drug-sensitive and multidrug-resistant P-glycoprotein-overexpressing leukemia cells, and normal leukocytes.

Cell type	Cell line	IC_50_ (µM)
Multiple myeloma	AMO1	1.8 ± 0.3
	JJN3	2.1 ± 0.7
	L363	2.5 ± 0.3
	MOLP8	2.5 ± 0.3
	OPM2	2.6 ± 0.6
	NCI-H929	2.7 ± 0.2
	RPMI8226	2.7 ± 0.4
	KMS11	2.8 ± 0.3
	KMS12BM	3.2 ± 0.2
Leukemia	CCRF-CEM	2.9 ± 0.0
	CEM/ADR5000	2.6 ± 0.2
Normal leukocytes	PBMCs- Donor 1	39.3 ± 1.4

**Table 3:** Validation of IC_50_ values using a narrower concentration range in four MM cell lines: AMO1, JJN3, OPM2, and L363 and in PBMCs collected from three different donors.

Cell type	Cell line	IC_50_ (µM)
Multiple myeloma	AMO1	1.8 ± 0.2
	JJN3	1.9 ± 0.4
	OPM2	2.3 ± 0.5
	L363	2.6 ± 0.6
Normal leukocytes	PBMCs- Donor 2	28.4 ± 3.8
	PBMCs- Donor 3	24.9 ± 7.8
	PBMCs- Donor 4	22.0 ± 4.5

The concentration of cynaropicrin needed to inhibit 50% of normal leukocytes differed from one donor to another and it ranged between 22.0 ± 4.5 µM and 39.3 ± 1.4 µM. The concentrations of cynaropicrin needed to inhibit half of the normal leukocytes were higher than the concentration needed to inhibit 50% of KMS12BM, the least sensitive cell line.

### Transcriptome-based expression profiles of cynaropicrin-treated AMO1 cells and Quantitative real time reverse transcription PCR (qRT-PCR)

**Fig. 2:**
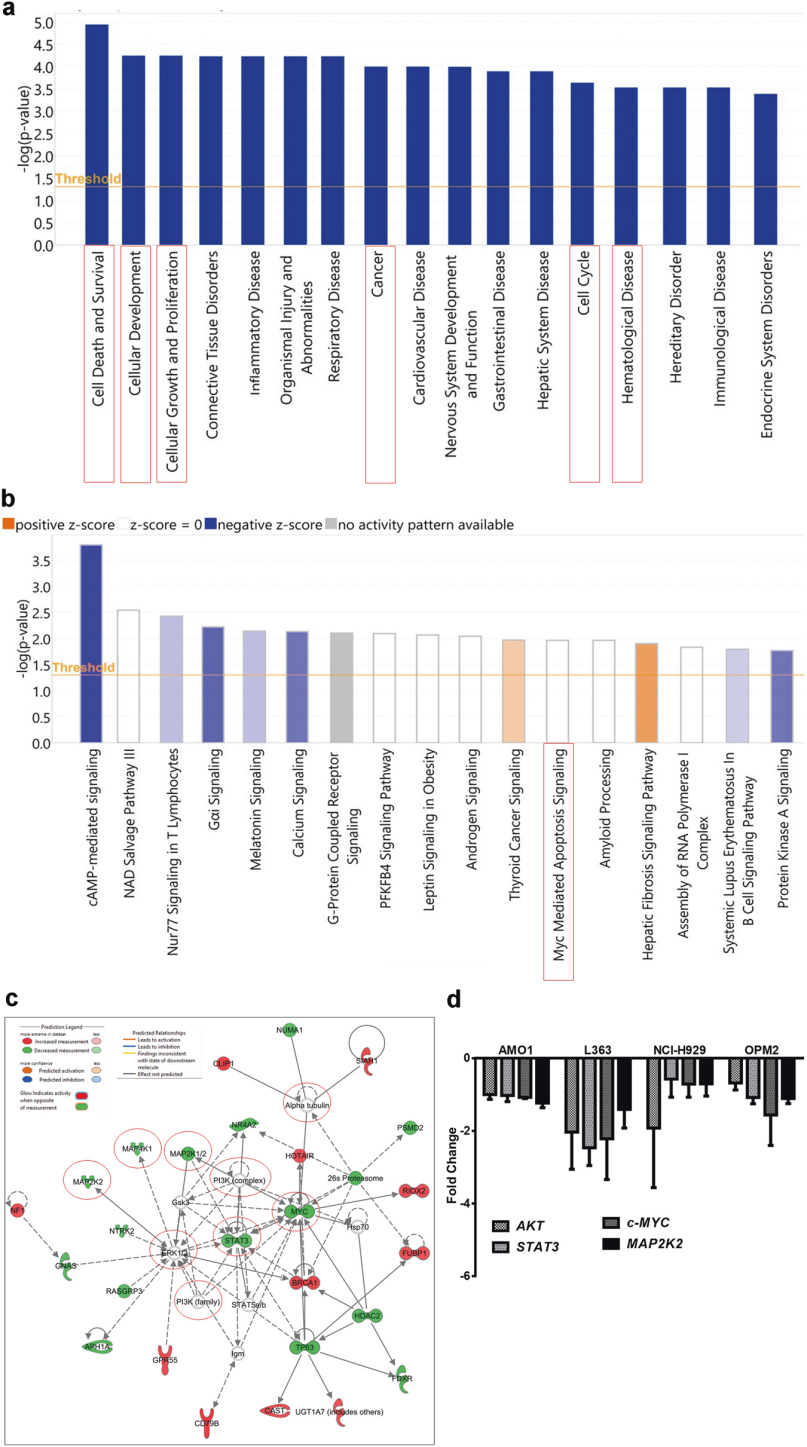
Ingenuity Pathway Analysis (IPA) using Affymetrix Clariom S human chips normalized microarray-based gene expression data. **a** Illustration of the top “biological functions” and “diseases”. **b** Canonical pathways. A cut-off threshold (–log (*P* value)) of 1.3 is considered as a default parameter. Only significant biological functions and diseases as well as canonical pathways with *P* < 0.05 are illustrated. **c** Prediction of a functional network by IPA of cynaropicrin-treated AMO1 cells. The red circles emphasize the downregulation of *c-MYC*, *STAT3*, and *MAPKs*. In addition, *PI3K* and *TUBA* appeared to be strongly involved within this pathway disturbed by cynaropicrin. Relative changes in gene expression profiles are displayed by a color-coding system: red represents upregulated genes and green depicts downregulated genes. **d** The expression levels of *AKT*, *STAT3*, *c-MYC*, and *MAP2K2* upon treatment of AMO1, L363, NCI-H929, and OPM2 with the corresponding IC_50_ value of cynaropicrin were determined by qRT-PCR. The X-axis represents the previously mentioned genes; however, the Y-axis shows the fold change.

AMO1 cells were treated with the IC_50_ concentration of cynaropicrin and subjected to mRNA expression profiling. Ingenuity Pathway Analyses software (IPA) (Qiagen, Hilden, Germany) was used to analyze differentially expressed genes compared to untreated samples. IPA reported several diseases and considerable cellular functions that might be altered by cynaropicrin, e.g., cell death and survival, cellular development, cellular growth and proliferation, cancer, cell cycle, and most importantly hematological disease (Fig. 2a). Additionally, IPA estimated many canonical pathways that might be affected by cynaropicrin. Among these pathways, c-Myc-mediated apoptosis signaling drew our attention (Fig. 2b). Upstream regulator analyses showed that *STAT3*, a transcription factor overexpressed in MM [51], was significantly deregulated by cynaropicrin (*P* = 1.96 × 10^−3^) (Table 4).

**Table 4:** Upstream regulators impaired by cynaropicrin.

Top Upstream Regulators	*P* value
*STAT3*	1.96 × 10^−3^
*MED1*	1.37 × 10^−2^
*MKNK1*	1.84 × 10^−2^
*SLC2A4*	1.84 × 10^−2^
*ATF4*	1.84 × 10^−2^

Furthermore, IPA suggested a network where both *STAT3* and c-*MYC*, a downstream proto-oncogene target of STAT3, were downregulated. Besides these two genes, MAPKs (*MAP2K2*, *MAP4K1*, *MAP2K1/2*), that play a crucial role in cell survival, proliferation, and death [52] were also downregulated by cynaropicrin (Fig. 2c). In addition to that, *PI3K* and *TUBA* appeared as connecting genes within this IPA pathway disturbed by cynaropicrin, rendering them eligible for further specific analyses.

Several genes (*AKT*, *STAT3*, c-*MYC*, and *MAP2K2*) were subjected to qRT-PCR, and their expression was normalized with the reference gene, *GAPDH*. Results were in line with the microarray analyses. In fact, treatment of AMO1 with cynaropicrin decreased the expression of *AKT*, *STAT3*, c-*MYC* and *MAP2K2* (Fig. 2d). To further validate this assumption, we detected the gene expression of *STAT3*, *AKT*, *c-MYC*, and *MAP2K2* in L363, NCI-H929, and OPM2. Our results were in line with that obtained in AMO1, and indeed *STAT3*, *AKT*, *c-MYC*, and *MAP2K2* gene expression was downregulated in L363, OPM2, NCIH929, and AMO1 upon treatment with cynaropicrin.

### Cynaropicrin repressed c-Myc upstream regulators as detected by Western blotting

**Fig. 3:**
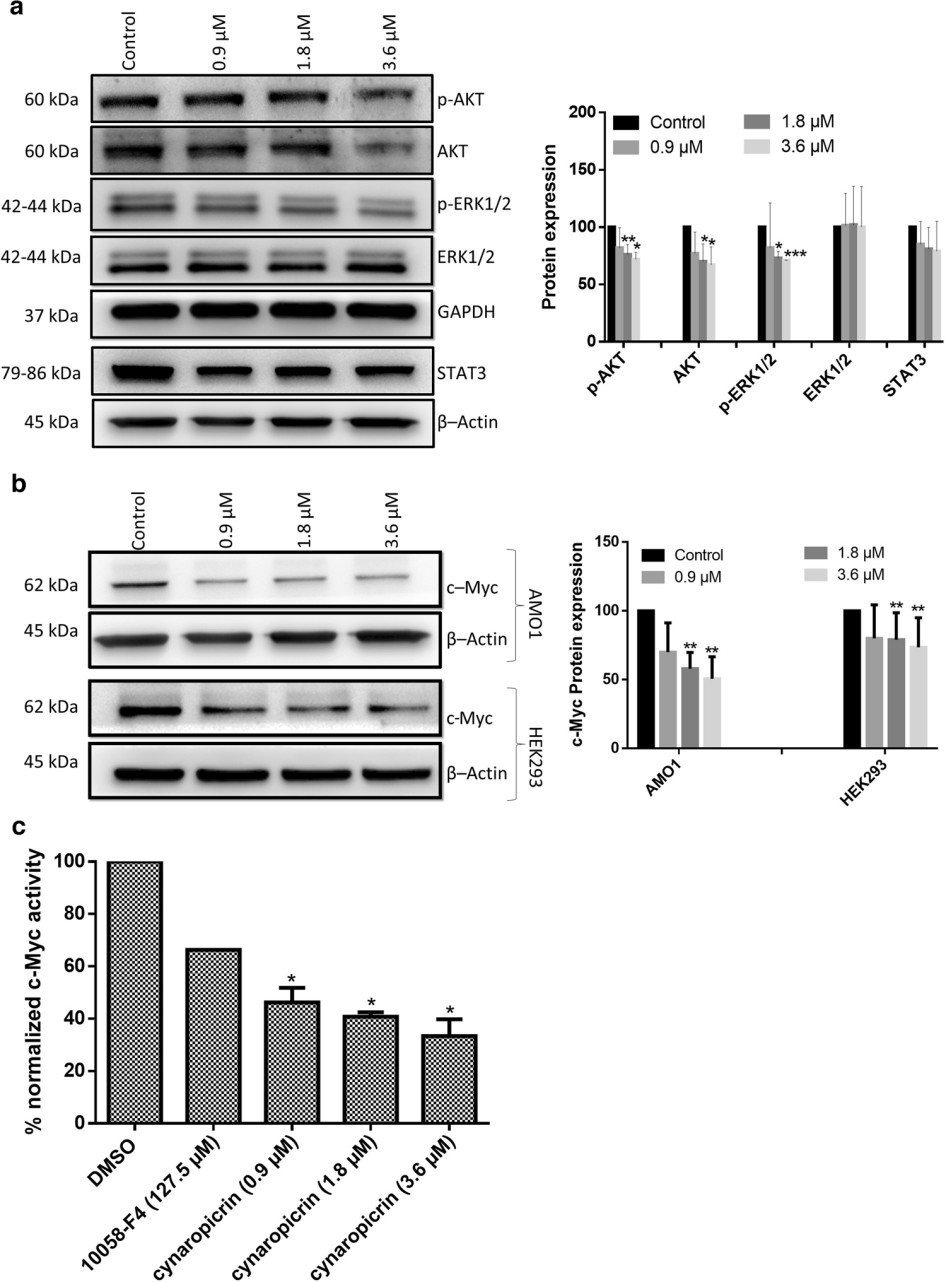
Cynaropicrin affected p-AKT, AKT, p-ERK1/2, ERK1/2, and c-Myc expression as well as c-Myc transcriptional activity. **a** Effect of cynaropicrin at different concentrations on the protein expression levels of p-AKT, AKT, p-ERK1/2, ERK1/2, and STAT3 in AMO1 cells as detected by Western blotting. **b** Effect of cynaropicrin on the protein expression levels of c-Myc in AMO1 and HEK293 cells as detected by Western blotting. The bar diagrams were obtained by calculating the mean value ± SD of three experiments performed at different time points. **c** Effect of cynaropicrin and the known c-Myc inhibitor 10058-F4 on c-Myc transcriptional activity as percentage of the normalized HEK293 cells transiently transfected with c-Myc luciferase reporter construct. The bar diagram was obtained by calculating the mean value ± SD of two independent experiments with three replicates each. *P < 0.05, **P < 0.01, ***P < 0.001 if compared to control sample.

IPA revealed that c-Myc upstream regulators were downregulated. These findings stimulated us to further evaluate the influence of cynaropicrin on signaling proteins that are upstream of c-Myc. Especially, ERK signaling, JAK2-STAT3, and PI3K-AKT which are strong mediators of MM cell proliferation, survival, anti-apoptosis, and resistance [53]. Our data showed that cynaropicrin treatment downregulated STAT3 in a dose-dependent manner. Furthermore, cynaropicrin significantly reduced the level of total AKT as well as its activated form p-AKT. However, cynaropicrin significantly downregulated p-ERK1/2 without affecting total ERK (Fig. 3a). Our findings indicated that cynaropicrin downregulated the protein level of c-Myc upstream regulators.

### Cynaropicrin inhibited c-Myc expression and transcriptional activity as detected by Western blotting and Myc reporter assay

mRNA expression profiles revealed that *c-Myc*-mediated apoptosis signaling was targeted by cynaropicrin and divulged a network where *c-Myc* was downregulated. It is well-known that MM rely strongly on c-Myc for growth and survival [54]. Therefore, it was crucial to determine whether cynaropicrin prohibited c-Myc expression and transcriptional activity. c-Myc expression was significantly downregulated in a dose-dependent way upon treatment with cynaropicrin (Fig. 3b).

In an attempt to discover if cynaropicrin inhibitory effect was not only restricted to c-Myc expression but could also include the inhibition of c-Myc transcriptional activity, human embryonic kidney 293 (HEK293) cells were transfected with a c-Myc reporter luciferase construct. These cells were treated with different cynaropicrin concentrations and with the known c-Myc inhibitor 10058-F4 [55] (F3680, Sigma–Aldrich, Darmstadt, Germany), respectively. Indeed, cynaropicrin inhibited c-Myc transcriptional activity in a dose-dependent manner. Additionally, low concentrations of cynaropicrin inhibited c-Myc activity more efficiently than the known inhibitor 10058-F4, indicating that cynaropicrin has a c-Myc inhibitory effect (Fig. 3c). To further validate that cynaropicrin inhibited c-Myc expression and transcriptional activity, we studied the expression of c-Myc in HEK293 cells. As expected, c-Myc expression significantly decreased upon cynaropicrin treatment (Fig. 3b).

### Cell cycle analyses of cynaropicrin-treated AMO1 cells

**Fig. 4:**
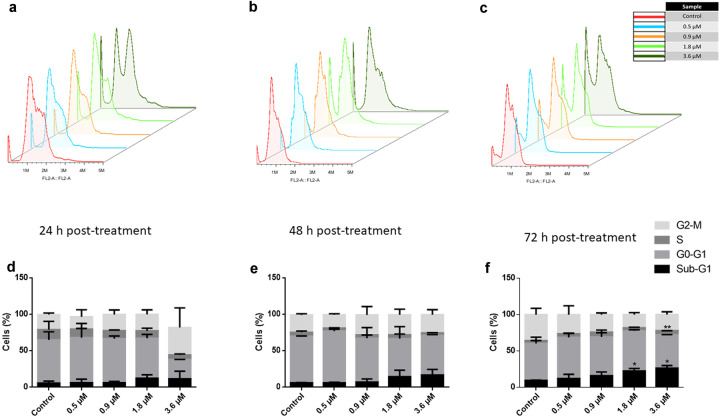
Cell cycle disturbance of AMO1 cells by cynaropicrin. DNA histograms of AMO1 cells subjected to several cynaropicrin concentrations for 24 h (**a**), 48 h (**b**), and 72 h (**c**). These histograms are generated by flow cytometry using the blue laser (*λ*/nm = 488) for excitation and by measuring the emission at λ/ nm =530. Bar diagrams representing the distribution of cynaropicrin-treated AMO1 cells in the different cell cycle phases after 24 h (**d**), 48 h (**e**), and 72 h (**f**). The percentage of cells in each phase is obtained by calculating the mean value ± SD of three experiments performed at different time points. *P < 0.05, **P < 0.01 if compared to the control sample.

Afterwards, the perturbations of the cell cycle caused by cynaropicrin were explored, given that the cell cycle was one of the cellular processes that appeared to be susceptible to change (Fig. 2a). The cell cycle status of AMO1 cells was evaluated after 24, 48, and 72 h incubation with various cynaropicrin concentrations (0.5, 0.9, 1.8, and 3.6 µM). After 24 h treatment, the fraction of AMO1 cells in the G_2_M phase and the sub G_0_G_1_ phase increased proportionally with increased cynaropicrin concentration. After 48 h and 72 h treatment, the portion of cells in the sub G_0_G_1_ phase noticeably increased with increased cynaropicrin concentration, implying that cynaropicrin induced cell death in AMO1 cells (Fig. 4).

### Cynaropicrin distorted the microtubule network

**Fig. 5:**
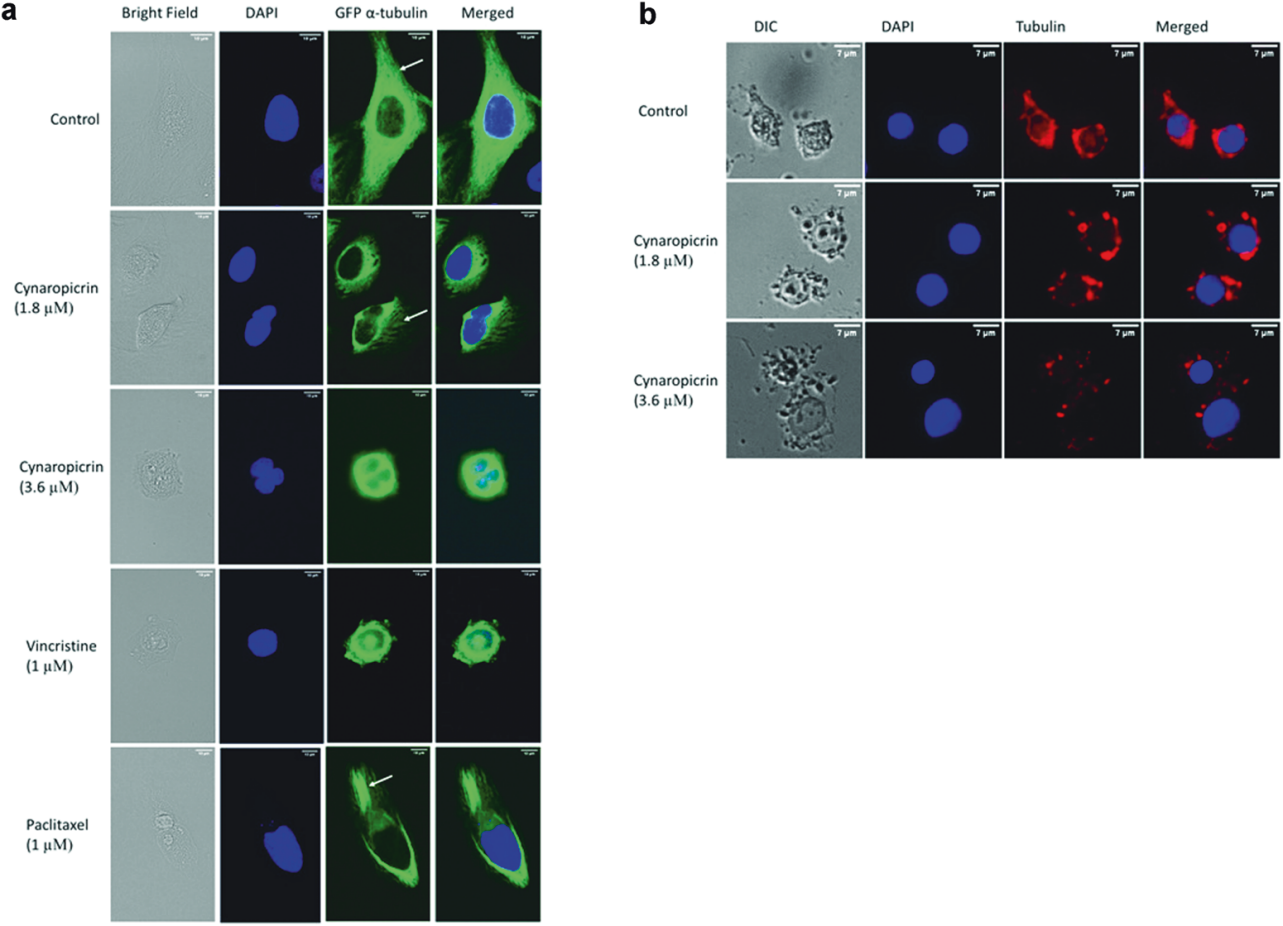
Disorganization of the microtubule distribution upon treatment with cynaropicrin. **a** U2OS cells stably express GFP-α-tubulin protein. Micrographs of U2OS cells fixed with 4% paraformaldehyde were photographed 24 h post-treatment with DMSO, 1.8 µM and 3.6 µM of cynaropicrin, 1 µM vincristine, or 1 µM paclitaxel. Cells nuclei were stained with DAPI (blue). The peripheral microtubule masses are represented by the white arrows. Images were taken with an AF7000 widefield fluorescence microscope at 40 × magnification (scale bars = 10 µm). **b** The living AMO1 cells were stained with Tubulin Tracker™ Deep Red. Micrographs of AMO1 cells were photographed 24 h post-treatment with DMSO, 1.8 µM and 3.6 µM of cynaropicrin. Cells nuclei were stained with Hoechst 33342 Nuclear Stain (blue). Images were taken with an AF7000 widefield fluorescence microscope at 40 × magnification (scale bars = 7 µm).

Microtubules are crucial for chromosome separation during mitosis, and it is likely that the inhibition of microtubule polymerization triggers mitotic arrest [56]. Taking into consideration that cynaropicrin induced G_2_M arrest after 24 h, we investigated whether cynaropicrin inhibited the tubulin network. U2OS cells stably expressing the α-tubulin-GFP fusion protein were exposed to different concentrations of cynaropicrin (1.8 and 3.6 µM) for 24 h in an effort to examine the drug’s impact on the microtubule network. The tubulin network in control cells was properly polymerized. This was evident by the substantial amount of tubulin that was dispersed throughout the cytoplasm and that resulted in a robust intracellular network. On the other hand, cynaropicrin treatment and vincristine treatment both led to a disorganized tubulin network. In contrast to paclitaxel-treated cells where the microtubule network seemed stiff, cynaropicrin and vincristine reduced the expansion of microtubules at the edges and increased the bulk of tubulin around the nucleus. In addition, if compared to untreated cells, cynaropicrin-treated cells had thinner microtubules at their extremities (Fig. 5a). Moreover, similar results were obtained in AMO1 cells exposed to 1.8 and 3.6 µM of cynaropicrin. Cynaropicrin treatment distorted the tubulin network of AMO1 cells if compared to control cells (Fig. 5b). These findings collectively showed that cynaropicrin, such as vincristine, inhibited the polymerization of the microtubule network.

### Cynaropicrin did not induce apoptosis or autophagy in AMO1 cells

**Fig. 6:**
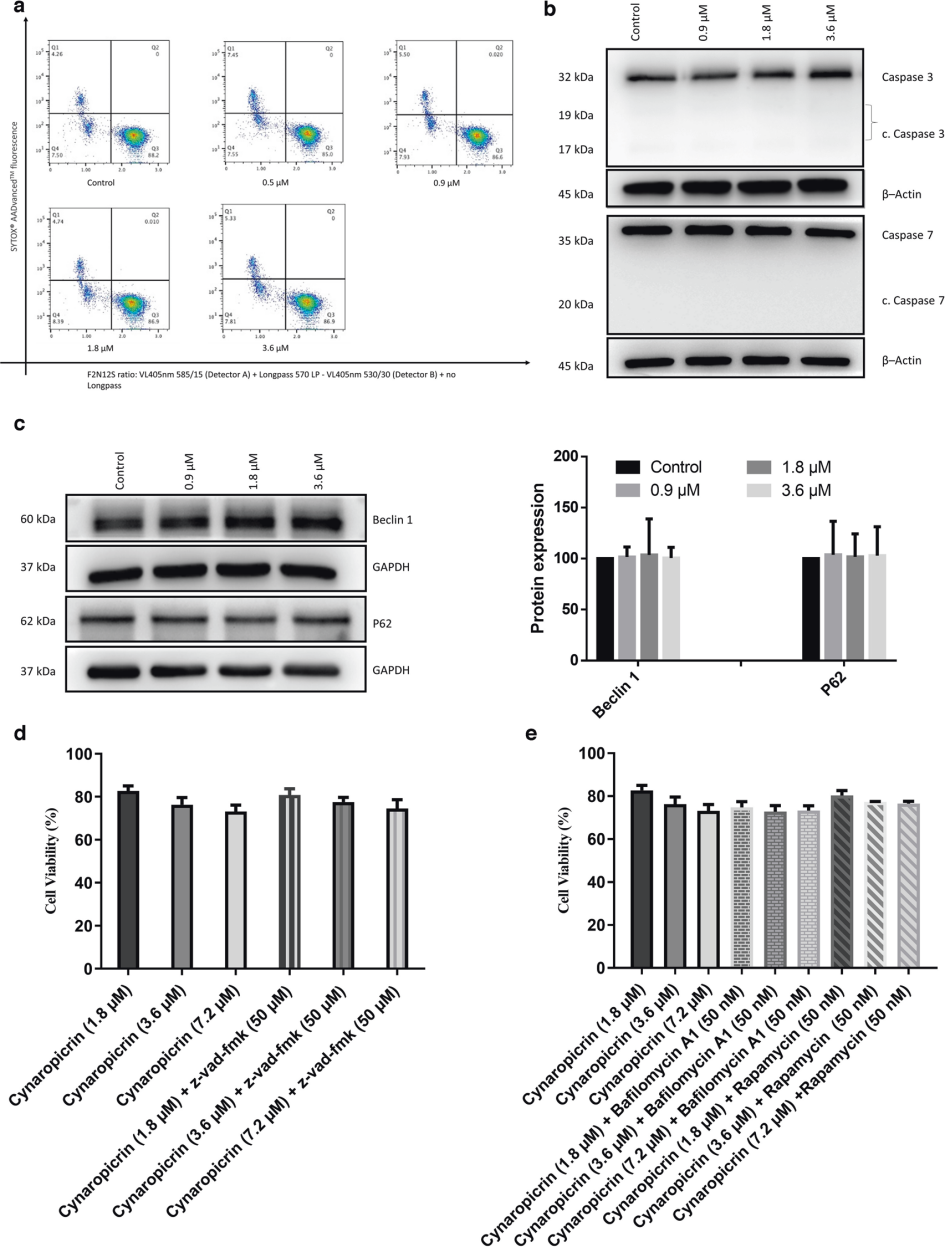
Evaluation of apoptosis and autophagy in AMO1 cells treated with different cynaropicrin concentrations (0.9, 1.8, and 3.6 µM) or DMSO for 48 h. **a** Cynaropicrin did not induce apoptosis in AMO1 cells. Apoptotic cells were measured using the Violet Ratiometric Membrane Asymmetry Probe/Dead Cell Apoptotic kit. Q1 depicts dead cells, Q3 illustrates living cells, and Q4 displays apoptotic cells. The numbers under Q1, Q3, and Q4 represent the percentage of cells. **b** Cynaropicrin prevented the cleavage of the executioner caspases (caspase 3 and caspase 7) as detected by Western blot. **c** Cynaropicrin did not affect the protein expression levels of the autophagy marker Beclin 1 and P62 as detected by Western blot. The bar diagram representing the protein expression level of Beclin 1 and P62 was obtained by calculating the mean value ± SD of three experiments performed at different time points. **d** Caspase inhibition by z-vad-fmk did not significantly alter the sensitivity of AMO1 to cynaropicrin. **e** The inhibition of autophagy by bafilomycin A1, as well as the induction of autophagy by rapamycin did not significantly alter the sensitivity of AMO1 to cynaropicrin.

Cell cycle analyses revealed that the portion of cells in the sub G_0_G_1_ phase noticeably increased with increased cynaropicrin concentration, indicating that cynaropicrin induced cell death in AMO1 cells. In an attempt to deliberate the mode of cell death induced by cynaropicrin, we examined the programmed cell death, apoptosis, as it is one of the probable mechanisms of action of cytotoxic drugs in cancer cells. The ratiometric probe F2N12S and the DNA-binding SYTOX™ AADvanced™ dye were used to measure cell membrane asymmetry (apoptotic cells) and cell permeability (dead cells), respectively. F2N12S and SYTOX™ AADvanced™ combined together discriminate between live, apoptotic, and dead cells. Our results revealed that only 7.5% of control cells experienced apoptosis and the percentage of apoptotic cells was the same even upon exposure to different cynaropicrin concentrations (Fig. 6a), implying that cynaropicrin did not induce apoptosis in AMO1 cells. To strengthen our findings, we further investigated apoptosis by detecting the apoptotic markers (caspase 3 and caspase 7) by Western blotting. Neither caspase 3 nor caspase 7 were cleaved by cynaropicrin treatment (Fig. 6b), suggesting that cynaropicrin induced a caspase independent cell death. Subsequently, we investigated the programmed non-apoptotic cell death autophagy by evaluating the autophagy specific marker (Beclin 1 and P62) by Western blotting (Fig. 6c). Cynaropicrin did not affect the expression of neither Beclin 1 nor P62, indicating that autophagy is not induced by cynaropicrin. Moreover, the pharmacological inhibition of caspase by z-vad-fmk, the inhibition of autophagy by bafilomycin A1, as well as the induction of autophagy by rapamycin did not significantly alter the sensitivity of AMO1 to cynaropicrin (Fig. 6d, e). So far, our data revealed that neither apoptosis nor autophagy was implicated in cynaropicrin-induced cell death, therefore further investigations must be performed to decipher the type of cell death induced by cynaropicrin.

### Cynaropicrin induced parthanatos as a novel cell death mode in AMO1 cells

**Fig. 7:**
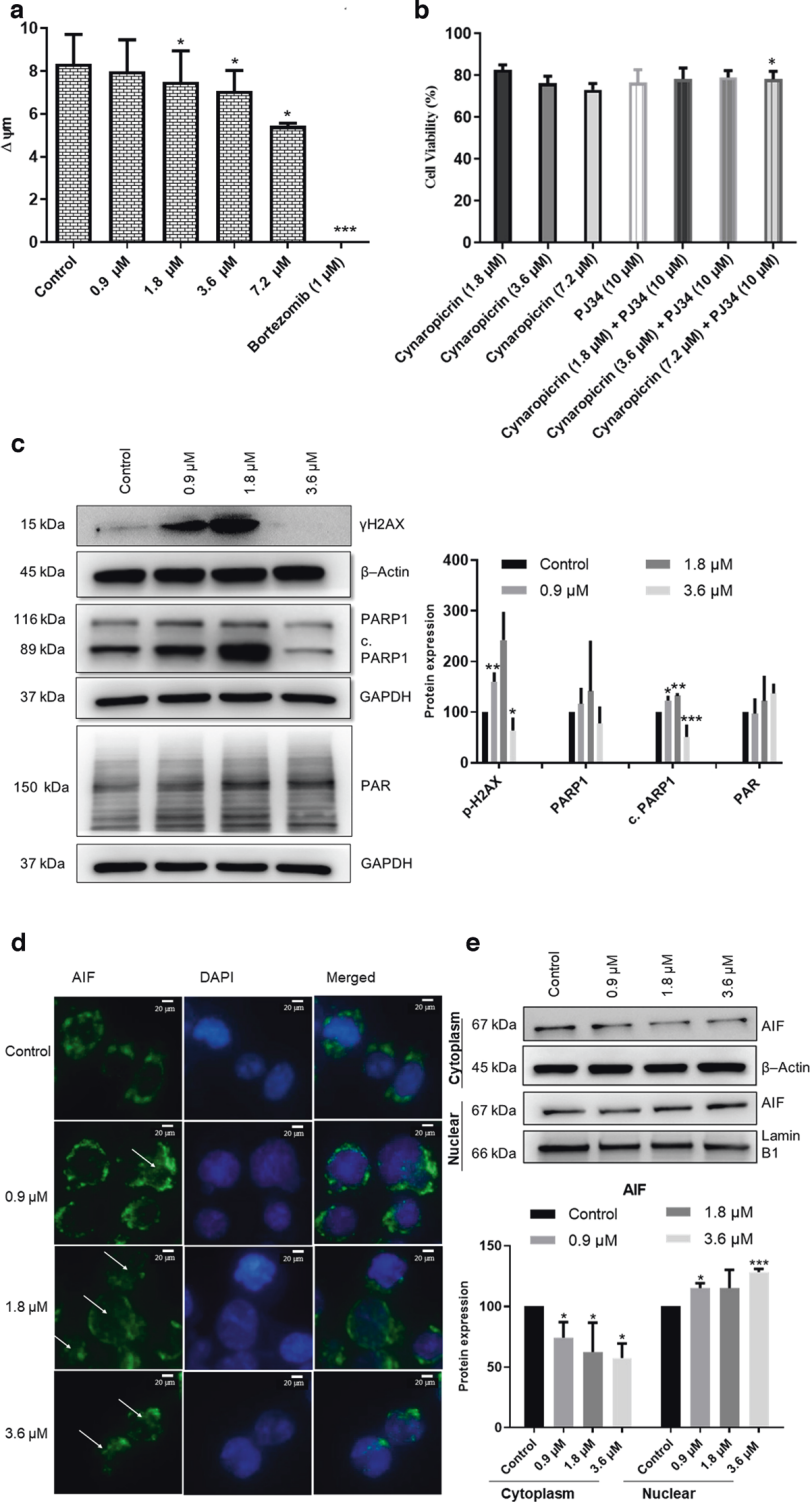
Investigation of parthanatos as a novel cell death in AMO1 cells treated with different cynaropicrin concentrations (0.9, 1.8, and 3.6 µM) or DMSO for 48 h. **a** Analysis of the mitochondrial membrane potential. The bar diagram represents the level of mitochondrial membrane potential (ΔΨm) which was discerned by JC-1-staining and measured by calculating the ratio of J-aggregates to J-monomers fluorescence intensity. **b** PARP inhibition by PJ34 increased AMO1 cell viability in presence of cynaropicrin. **c** Effect of cynaropicrin on the expression levels of several proteins involved in the parthanatic cell death as detected by Western blot. **d** Confocal microscopy images of apoptosis inducing factor (AIF) immunofluorescence showing the translocation of AIF from the cytoplasm to the nucleus in AMO1 cells treated with cynaropicrin for 48 h. The arrows highlight the release of AIF into the cytoplasm. Images were taken at 50 × magnification (scale bars = 20 µm). **e** Effect of cynaropicrin on the expression level of AIF in the cytoplasm and the nucleus as detected by Western blot. Bar diagrams were obtained by calculating the mean value ± SD of three experiments performed at different time points. *P < 0.05, **P < 0.01, ***P < 0.001 if compared to control sample.

It is generally known that mitochondria release death factors whenever a cell is under stress [57]. Therefore, to evaluate if the mitochondria underwent critical changes upon cynaropicrin treatment, we investigated the changes of the inner transmembrane potential Δψm in cynaropicrin-treated AMO1 cells using JC-1 probe. Cynaropicrin treatment led to a significant dose-dependent loss of the mitochondrial membrane potential. Bortezomib used as a positive control absolutely induced mitochondrial membrane depolarization (Fig. 7a).

Our findings so far implicated that cynaropicrin-induced cell death was mitochondrial-linked, though caspase-independent. Because apoptosis-inducing factor (AIF) triggers cell death in a caspase-independent way and because it is one of the most eminent parthanatic biomarkers [58], we next investigated parthanatos as a potential novel mode of cell death induced by cynaropicrin. One of the hallmarks of parthanatos is the overactivation of poly (ADP-ribose) polymerase 1 (PARP1) [58]. Therefore, we determined by Western blotting the level of PAR polymer, which is produced if PARP-1 is activated [59]. Our data revealed that PAR polymer expression clearly increased in AMO1 cells after being exposed to different cynaropicrin concentrations (Fig. 7c). Next, we examined cynaropicrin-induced changes in PARP1 protein level. Western blotting results revealed different PARP1 isoforms. The full-length PARP1 (116 kDa) indicated PARP1 activity, while the 89 kDa band denoted PARP1 cleavage [60]. Although a portion of PARP1 was cleaved following cynaropicrin treatment, the expression of PARP1 increased if AMO1 cells were treated with 0.9 and 1.8 µM of cynaropicrin, however, if treated with 3.6 µM of cynaropicrin the expression of PARP1 as well as c. PARP1 dropped (Fig. 7c).

Considering that DNA damage is a crucial initiator of parthanatos and that PARP-1 is triggered in response to various kinds of DNA damage [61], we then tested the protein level of phospho-Histone H2AX (Ser139) known as γH2AX and as a marker of DNA double strand breaks. Western blot results showed that γH2AX obviously increased when AMO1 cells were treated with 0.9 and 1.8 µM of cynaropicrin, however, if treated with 3.6 µM of cynaropicrin γH2AX expression diminished (Fig. 7c), indicating that cynaropicrin prompted DNA damage. Subcellular protein fractionation revealed that cynaropicrin treatment led to a dose-dependent substantial decrease in cytoplasmic AIF, which was accompanied by a significant increase in nuclear AIF (Fig. 7e). Confocal microscopy further validated that AIF translocated from the cytoplasm into the nucleus following cynaropicrin treatment (Fig. 7d). The pharmacological inhibition of PARP by PJ34 restored the cell viability of AMO1 but not to 100% due to the cytotoxic activity of PJ34 (Fig. 7b). All in all, these findings demonstrated that cynaropicrin triggered DNA damage, leading to the hyperactivation of PARP1 and the accumulation of PAR polymer in the cytoplasm, which in turn caused mitochondrial depolarization and AIF nuclear translocation.

### Anticancer activity of cynaropicrin in vivo

**Fig. 8:**
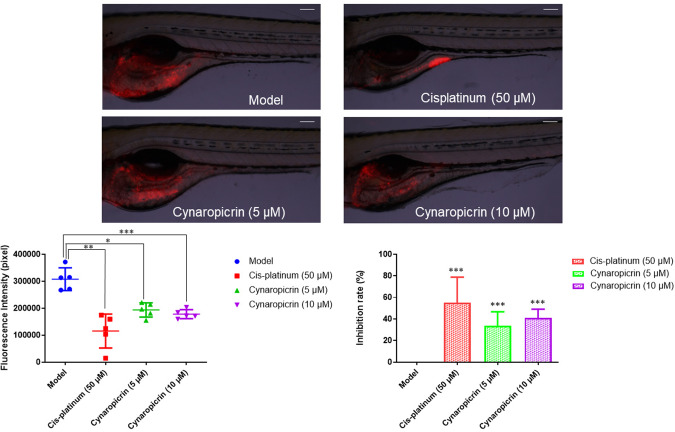
Acute cytotoxicity examination (fluorescence intensity and inhibition rate) of cynaropicrin in CCRF-CEM xenograft tumor zebrafish model (*n* = 5 zebrafish). The red fluorescence highlights CCRF-CEM tumor mass. Images were taken at 60× magnification (scale bars = 100 µm). *P < 0.05, **P < 0.01, ***P < 0.001 if compared to the model.

The antitumor activity of cynaropicrin has been investigated in vivo using golden hamsters or murine models [29]. In our study, a CCRF-CEM xenograft tumor model was developed in larvae zebrafish and fluorescence intensity was assessed 24 h after treatment to determine the inhibition rate of the tumor. The positive control cis-platinum effectively repressed the fluorescence intensity of CCRF-CEM tumor if compared to the untreated control group (Fig. 8). Similarly, cynaropicrin inhibited CCRF-CEM tumor growth with an inhibitory rate of 33.7% at low concentration (5 µM), and this inhibitory rate increased in a dose-dependent manner to reach 41.01% at higher concentration (10 µM). Our findings revealed that cynaropicrin exhibited an anti-tumor activity in a larvae zebrafish model.

## Discussion

Despite recent improvements in medical science that quadrupled the survival rate of MM patients over the past 40 years, this disease is practically uncurable due to the outgrowth of resistant subclones, upon receiving the first-line treatment [62]. Therefore, researchers are nowadays looking for alternative therapies to overcome resistance. Herein, natural products provided a variety of compounds that were applied in pharmacology and medicine for their promising impact on the reduction and the suppression of tumor development [63]. Cynaropicrin, the major ingredient of the edible parts of the artichoke plant, was applied as a safe diet for ages. Currently, the noteworthy anticancer activity of cynaropicrin captivated the attention of several researchers based on two important characteristics: its tiny size and water solubility which increase its membrane permeability, rendering therapeutic injections easy to formulate [29]. Cynaropicrin possessed a strong anti-proliferative activity against leukocyte cancer cells [36], and chronic myeloid leukemia cells [63]. Yet so far, no studies have been conducted on MM. Hence, the aim of the present work was to investigate the cytotoxicity and the mode of action of cynaropicrin in MM in vitro and in vivo.

Therefore, we examined a panel of 9 MM cell lines in addition to the multidrug-resistant CEM/ADR5000 T-ALL cells and their sensitive counterpart, CCRF-CEM T-ALL cells. All cell lines were impeccably sensitive towards cynaropicrin, with AMO1 being the most sensitive cell line. Hence, we examined the mechanisms of action of cynaropicrin in AMO1 cells. CEM/ADR5000 cells overexpressing the ABC-transporter P-glycoprotein are known to efflux chemotherapeutic drugs out of the cell, leading to chemotherapy failure [64]. Our data showed that CEM/ADR5000 cells exhibited comparable sensitivity to cynaropicrin as their sensitive sublines. Additionally, the resistance ratio was only 0.9, which is considerably lower than that of doxorubicin, a standard chemotherapeutic drug with a degree of cross-resistance significantly higher than 1000 [65]. These findings implied that P-glycoprotein does not intervene in resistance to cynaropicrin. The doses of cynaropicrin needed to inhibit the growth of normal leukocytes by 50% (22.0 ± 4.5 µM ≤ IC_50_ ≤ 39.3 ± 1.4 µM) were much greater than in all examined leukemia and MM cell lines. This implies that the doses of cynaropicrin required to inhibit the proliferation of leukemia and MM cells could be attained without damaging healthy cells.

Microarray gene expression profiles were applied to evaluate the effects of cynaropicrin on AMO1 transcriptional activity. Various differentially expressed genes were identified in cynaropicrin treated cells. IPA elucidated a network where c-*MYC*, *STAT3*, *MAP2K1/2*, *MAP2K2*, and *MAP4K1* were downregulated. Furthermore, the *PI3K* complex and *TUBA* were targeted by cynaropicrin. Remarkably, several diseases and canonical pathways were affected by cynaropicrin, especially hematological diseases and *c-Myc*-mediated apoptosis signaling. Upstream target analyses proposed *STAT3* as potential upstream target of cynaropicrin. STAT3 is a transcription factor known to be basically activated in several human cancer cells as well as hematological tumors. Another significant signal transduction pathway in B-cell neoplasms involved AKT and PI3K. In fact, aberrant activation of PI3K pathway prevailed in hematological diseases. Previous studies demonstrated the critical roles of STAT3 and PI3K in survival, growth, and chemotherapy resistance of plasma-cells and B-cells neoplasms [66]. Yet, MAPK-pathway was also among the prominent upregulated signaling pathways in MM [67].

C-Myc is a transcription factor known to play a crucial role in several cellular processes as transcription, translation, proliferation, apoptosis, and metabolism [68]. It was firstly discovered as the homolog of an avian retrovirus and as being upregulated in Burkitt’s Lymphoma [69–71]. C-Myc controls the progression of the cell cycle, therefore the inhibition of c-Myc led to cell cycle arrest and impairment of cell cycle progression in human myeloid and lymphoid cells [72]. C-Myc deregulation was associated with MM, therefore any therapeutic strategies targeting it would be valuable in treating B-cells malignancy. However, therapies against c-Myc were and, by some means, are still challenging for the absence of a nuclear localization, a defined 3-D structure, and an enzymatic pocket. Despite these drawbacks, many studies showed the potential impact of c-Myc inhibition. C-Myc is a downstream target of the MAPK, STAT3, and PI3K pathways. Therefore, it is foreseen as the master integrator and regulator of signaling networks associated with cancer [67]. Based on microarray analyses performed here, we assumed that cynaropicrin exposure inhibited STAT3/AKT/ERK signaling and c-Myc, engendering the cynaropicrin anti-proliferative activities in MM. This assumption was validated on the RNA level through RT-qPCR, on the protein level through immunoblotting, and by performing a c-Myc reporter assay. RT-qPCR revealed that c-*MYC* was downregulated upon treatment with cynaropicrin. Moreover, cynaropicrin remarkably inhibited c-Myc expression and exerted a strong inhibition of c-Myc DNA binding activity. Interestingly, this inhibitory effect was much more significant than that of the known c-Myc inhibitor 10058-F4. Furthermore, the concentrations of cynaropicrin needed to inhibit c-Myc were extremely lower than that of 10058-F4. To the best of our knowledge, we are the first to report that cynaropicrin devastated MM cells by inhibiting c-Myc, implying a novel mode of action of cynaropicrin as an anti-tumor drug.

With an eye toward investigating how cynaropicrin inhibited c-Myc expression as well as activity, we checked the effect of cynaropicrin on upstream regulators of c-Myc. A previous study revealed that cynaropicrin decreased various cellular malignant characteristics in melanoma through the suppression of ERK1/2 and NF-κB activity [31]. Butturini et al. also described that cynaropicrin regulated STAT3 function [73]. Moreover, cynaropicrin exhibited anti-proliferative and apoptotic activities in lung cancer cells through the inactivation of the EGFR/AKT signaling pathway [74]. Our data were in line with those findings as cynaropicrin exerted significant inhibitory effects on STAT3, AKT, and ERK pathways. The fact that the basal level of AKT was downregulated might imply that cynaropicrin affects its synthesis rate at any point of transcription and translation. STAT3, AKT, and ERK pathways play a major role in controlling the stability, the accumulation, as well as the transcriptional activity of c-Myc [75]. Consequently, the inhibition of STAT3, PI3K, and MAPK pathways might downregulate c-Myc. Our data revealed that reduced c-Myc expression upon treatment with cynaropicrin was strongly associated with the inhibition of STAT3, AKT, and ERK1/2. These findings further validated the key role of those pathways in c-Myc regulation.

We also investigated the expression of c-Myc in HEK293 cells to confirm that cynaropicrin reduced c-Myc transcriptional activity. As predicted, a considerable downregulation of c-Myc was observed.

Afterward, cell cycle arrest was examined by analyzing the distribution of cynaropicrin-treated AMO1 cells through the sub-G_0_G_1_, G_0_G_1_, S, and G_2_M phases, using flow cytometry, after 24 h, 48 h, and 72 h incubation. Cells were assembled in the G_2_M phase 24 h after cynaropicrin treatment. Thus, mediators of the G_2_M phase may be affected by cynaropicrin. Therefore, we concentrated on examining cynaropicrin’s effect on the microtubule cytoskeleton. Our results were in line with previous studies demonstrating that cynaropicrin promoted G_2_M arrest in lung epithelial A549 cancer cells, in human anaplastic thyroid cancer, and in breast MDA-MB-231 cancer cells [32, 33, 76]. The inappropriate entry of cells in the mitotic process is known as mitotic catastrophe, a process that recognizes mitotic failure and reacts by sending a cell into an irrevocable antiproliferative death [77]. This could assist in understanding the increase in the sub-G_0_G_1_ phase with increased cynaropicrin concentration after 24 h, 48 h, and 72 h treatment. Microtubules are extremely active cytoskeletal elements that are crucial for a variety of cellular processes, including cell division, vesicle transport, and intracellular structure. Microtubule targeting agents suppress the dynamic of the spindle microtubules, which causes mitosis to slow down or stop. This block in the G_2_/M phase results in cell death [78]. Thereafter, we studied the impact of cynaropicrin on the microtubule cytoskeleton through confocal microscopy of U2OS cells stably expressing α-tubulin-GFP and by visualizing the stained tubulin network of AMO1 cells. Indeed, the microtubule organization was aberrant in both cell lines. Similarly to vincristine, cynaropicrin reduced the density of microtubules at the edges and increased the mass of tubulin around the nucleus. Moreover, cynaropicrin-treated cells had thinner microtubules at their extremities, if compared to untreated cells. Mastimoto et al. recently identified tubulin as a binding protein of cynaropicrin. However, we demonstrated in this study for the first time that cynaropicrin disassembled the microtubule network, and this could be an explanation of its cytotoxicity [79]. Transfected U2OS human cells were used as a model to detect the effect of cynaropicrin on tubulin network. Adherent cells are known to have a well polymerized tubulin network, however, MM are small suspension cells with a very tiny tubulin network, rendering it hard to visualize and making U2OS cells a much better model to observe effects on microtubules.

Sesquiterpene lactones are known to be potent inducers of cytotoxicity by initiating apoptosis [36]. Therefore, we investigated whether cynaropicrin-induced cytotoxicity in AMO1 cells is related to apoptosis. Our results indicated that cynaropicrin did not induce apoptosis as evident by the absence of changes in cell membrane asymmetry and cell permeability in cynaropicrin treated AMO1 cells compared to untreated cells after 48 h incubation, the absence of activated caspase 3 and caspase 7 that are known as apoptotic markers, and the unaffected sensitivity of AMO1 to cynaropicrin upon caspase inhibition. These results suggested that cynaropicrin caused cell death in a caspase-independent manner. We next addressed whether cynaropicrin-induced cytotoxicity is linked to autophagy. The fact that the expression of Beclin1 and P62, two autophagy markers, did not change after treatment and the combination of cynaropicrin with either bafilomycin A1 or rapamycin did not significantly alter the sensitivity of AMO1 to cynaropicrin refuted this assumption as well. Unexpectedly, our findings were contradictory to previously published research that suggested that cynaropicrin-induced cytotoxicity is associated to either apoptosis or autophagy [76, 80, 81]. In an attempt to decipher the mode of cell death induced by cynaropicrin in more detail, we studied the effect of cynaropicrin on the mitochondrial membrane potential as mitochondria are the cellular powerhouse and important regulators of cell death [82]. Our results indicated that cynaropicrin treatment indeed resulted in a significant loss of the mitochondrial membrane potential. Thus far, our findings suggested that cynaropicrin-induced cell death was non-apoptotic, mitochondrial-linked, though caspase independent. Given that AIF is a potential caspase-independent cell death effector and since it is one of the most prominent parthanatic biomarkers [58], we further focused on parthanatos as a potential novel cell death modality induced by cynaropicrin. During parthanatos, excessive DNA damage induces extensive PAR polymer production by PARP1 hyperactivation. The translocation of PAR into the cytoplasm alters the activity and the location of cytoplasmic proteins, resulting in the translocation of AIF from the mitochondria to the nucleus and subsequently to cell death [58, 59]. DNA damage is a crucial initiator of parthanatos. Therefore, we tested the level of marker protein γH2AX by Western blotting. Our results showed that γH2AX clearly increased if AMO1 cells were treated with cynaropicrin, indicating that cynaropicrin initiated DNA damage. As DNA damage is a cognitive factor that induces PARP-1 activation, we consequently assumed that cynaropicrin-induced DNA damage was implicated in the parthanatic cell death. In order to attract DNA repair machinery to DNA defects, the nuclear protein PARP1, activated by adhering to DNA lesions, catalyzes the poly(ADP-ribosylation) (PARylation) of nuclear acceptor proteins, along with PARP1 itself [59]. PARP1 activation was detected by the increase in its expression upon treatment with cynaropicrin. However, at high concentration (3.6 µM) the expression of PARP1 as well as cleaved PARP1 dropped off. This drop is accompanied by an increase in PAR polymer. The decrease in PARP1 expression could be explained by the fact that PARP enzymes regulate the function of AIF by adding ADP-ribose polymers (PAR) to it while utilizing NAD^+^ as a substrate [83, 84]. The binding of PAR to AIF was essential for AIF translocation from the mitochondria to the nucleus, and PAR binding was crucial for AIF to promote parthanatos [85]. Surprisingly, a partial cleavage of PARP1 was observed, although caspase 3 was not activated. It has been reported that PARP1 could be cleaved by several proteases other than caspases [86]. For instance, granzyme B, a serine protease secreted from natural killer cells and cytotoxic T cells, is involved in eradicating cancer cells. Granzyme B and caspase 3 both cleave PARP1 at the same site (Asp-Glu-Val-Glu), generating the 89 kDa PARP1 fragment. The 89 kDa PARP1 fragment acts as a cytoplasmic PAR carrier to improve AIF-induced DNA fragmentation [59]. Thus, our results suggest a similar mechanistic function. AIF, a target flavoprotein of PAR polymer during parthanatos, is normally located in the mitochondria. The excessive accumulation of PAR polymer resulted in the loss of the mitochondrial membrane potential. In response to such stimuli, AIF translocated from the mitochondria into the nucleus, and subsequently induced cell death [61]. Consistent with the hallmarks of parthanatic cell death, we also found that AIF translocated from the mitochondria to the nucleus after treatment of AMO1 cells with cynaropicrin. The level of total γH2AX decreased at (3.6 µM) and this decrease is accompanied by an increase in nuclear AIF. The reduction of total γH2AX could be explained by the fact that γH2AX interacts with AIF in the nucleus to generate a DNA-degrading complex that regulate DNA degradation [87]. The combination of cynaropicrin with PJ34 increased the number of living cells, and thus supporting the parthanatic cell death. To the best of our knowledge, we are the first to prove that cynaropicrin induced the novel mode of cell death parthanatos in MM, and to also confirm this novel cell death modality in MM.

Several researchers confirmed the antitumor efficacy of cynaropicrin in vivo using different animal models [35, 88]. Recently, the zebrafish model turned out to be a suitable in vivo model for drug discovery as well as toxicity assessment [67, 89]. In the present study, we thus proved that cynaropicrin reduced tumor growth in vivo using a CCRF-CEM xenograft tumor zebrafish model.

In summary, the insights acquired in this investigation suggest that cynaropicrin is a natural product that effectively reduces tumor growth in zebrafish via the novel parthanatic cell death. Cynaropicrin also causes potential cytotoxicity in vitro by inhibiting c-Myc, and subsequently STAT3, AKT, and ERK1/2, and by suppressing the tubulin network. Our findings revealed that the potential therapeutic value of cynaropicrin is the outgrowth of parthanatos, a novel mode of cell death.

### Supplementary information

Supplementary table 1

Supplementary Fig. 1
