# Supplementary material for: Cynaropicrin disrupts tubulin and c-Myc-related signaling and induces parthanatos-type cell death in multiple myeloma
Source: Acta Pharmacol Sin. 2023 Jun 21;44(11):2265–81. doi: 10.1038/s41401-023-01117-3 (PMC10618500; doi:10.1038/s41401-023-01117-3)
Supplement: Supplementary file 1 — Supplementary table 1 [file 41401_2023_1117_MOESM1_ESM.docx]

Supplementary information

Supplementary table 1: Top upregulated and downregulated genes by cynaropicrin.

| **Top upregulated genes** | **Expression fold change** | **Top downregulated genes** | **Expression fold change** |
| --- | --- | --- | --- |
| *GPRC5D* | 1.548 | *TP63* | -1.586 |
| *HNRNPA2B1* | 1.454 | *NR4A2* | -1.439 |
| *NPIPB15 (includes others)* | 1.404 | *AGBL5* | -1.400 |
| *RESF1* | 1.404 | *POLRD1* | -1.380 |
| *CD79B* | 1.357 | *MAP2K2* | -1.376 |
| *SCARNA11* | 1.352 | *RRBP1* | -1.357 |
| *HAPLN1* | 1.333 | *MAGEC1* | -1.357 |
| *FTLP2* | 1.297 | *SULF2* | -1.343 |
| *PRMD15* | 1.297 | *SIPA1* | -1.343 |
| *SNORA77* | 1.297 | *APH1A* | -1.343 |
