# Supplementary material for: Cynaropicrin disrupts tubulin and c-Myc-related signaling and induces parthanatos-type cell death in multiple myeloma
Source: Acta Pharmacol Sin. 2023 Jun 21;44(11):2265–81. doi: 10.1038/s41401-023-01117-3 (PMC10618500; doi:10.1038/s41401-023-01117-3)
Supplement: Supplementary file 2 — Supplementary Fig. 1 [file 41401_2023_1117_MOESM2_ESM.docx]

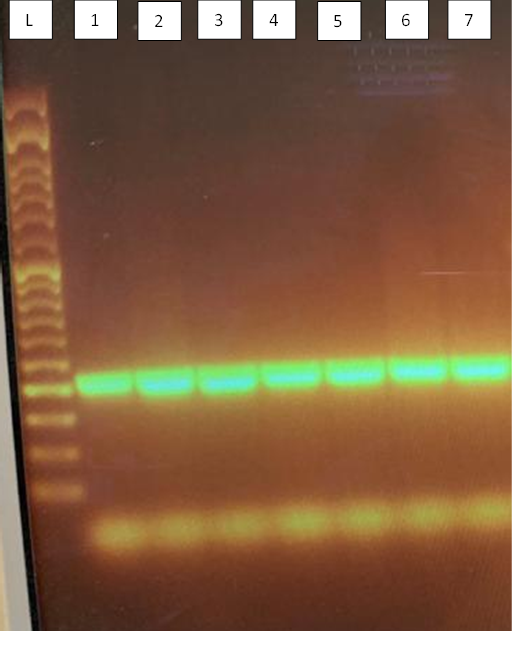


500 bp bp

200 bp bp

Supplementary Fig. 1: Examination of mycoplasma in 6 MM cell lines.

L: DNA ladder Lane 4: KMS12BM

Lane 1: control reaction without template Lane 5: MOLP8

Lane 2: AMO1 Lane 6: NCIH929

Lane 3: KMS11 Lane 7: RPMI8226
